# Dosimetric Optimization and Commissioning of a High Field Inline MRI-Linac

**DOI:** 10.3389/fonc.2020.00136

**Published:** 2020-02-14

**Authors:** Urszula Jelen, Bin Dong, Jarrad Begg, Natalia Roberts, Brendan Whelan, Paul Keall, Gary Liney

**Affiliations:** ^1^Department of Medical Physics, Ingham Institute for Applied Medical Research, Liverpool, NSW, Australia; ^2^Liverpool Cancer Therapy Centre, Radiation Physics, Liverpool, NSW, Australia; ^3^School of Medicine, University of New South Wales, Sydney, NSW, Australia; ^4^Centre for Medical Radiation Physics, University of Wollongong, Wollongong, NSW, Australia; ^5^Sydney Medical School, ACRF Image X Institute, University of Sydney, Sydney, NSW, Australia

**Keywords:** MRI-linac, commissioning, beam characterization, dosimetry, magnetic field

## Abstract

**Purpose:** Unique characteristics of MRI-linac systems and mutual interactions between their components pose specific challenges for their commissioning and quality assurance. The Australian MRI-linac is a prototype system which explores the inline orientation, with radiation beam parallel to the main magnetic field. The aim of this work was to commission the radiation-related aspects of this system for its application in clinical treatments.

**Methods:** Physical alignment of the radiation beam to the magnetic field was fine-tuned and magnetic shielding of the radiation head was designed to achieve optimal beam characteristics. These steps were guided by investigative measurements of the beam properties. Subsequently, machine performance was benchmarked against the requirements of the IEC60976/77 standards. Finally, the geometric and dosimetric data was acquired, following the AAPM Task Group 106 recommendations, to characterize the beam for modeling in the treatment planning system and with Monte Carlo simulations. The magnetic field effects on the dose deposition and on the detector response have been taken into account and issues specific to the inline design have been highlighted.

**Results:** Alignment of the radiation beam axis and the imaging isocentre within 2 mm tolerance was obtained. The system was commissioned at two source-to-isocentre distances (SIDs): 2.4 and 1.8 m. Reproducibility and proportionality of the dose monitoring system met IEC criteria at the larger SID but slightly exceeded it at the shorter SID. Profile symmetry remained under 103% for the fields up to ~34 × 34 and 21 × 21 cm^2^ at the larger and shorter SID, respectively. No penumbra asymmetry, characteristic for transverse systems, was observed. The electron focusing effect, which results in high entrance doses on central axis, was quantified and methods to minimize it have been investigated.

**Conclusion:** Methods were developed and employed to investigate and quantify the dosimetric properties of an inline MRI-Linac system. The Australian MRI-linac system has been fine-tuned in terms of beam properties and commissioned, constituting a key step toward the application of inline MRI-linacs for patient treatments.

## Introduction

Limitations of image-guidance based on MV and kV radiation beams prompted development of systems combining linear accelerators and Magnetic Resonance Imaging (MRI) scanners (1). These hybrid systems, MRI-linacs, offer superior soft tissue contrast for visualization of the tumor and of the organs at risk which can be used for daily plan adaptation and/or real-time imaging during the treatment dose delivery. Four MRI-linac designs exist to date, employing a range of magnetic field strengths and two beam-to-magnetic-field orientations: *perpendicular* (or *transverse*) and *parallel* (or *inline*) and have been recently reviewed by Liney et al. (2). Two transverse systems: Unity (Elekta, UK) (3) and MRIdian (Viewray, USA) (4) are now available commercially and used clinically, while the two inline designs: Aurora RT (MagnetTx Oncology Solutions, Canada) (5) and Australian MRI-Linac (6) are at the research prototype stage.

Unique characteristics of these systems and mutual interactions between their components pose specific challenges for their commissioning and quality assurance. The foremost is the compatibility of the dosimetric equipment with the magnetic field due to the presence of ferrous materials or unscreened mechanical or electrical components. Furthermore, the hybrid nature of the MRI-linac treatment units requires the assessment of their concurrent functionalities, for instance dose deposition during imaging, congruence of the imaging and radiation isocentres, RF interference or gantry movement effect on the magnetic field homogeneity (7). And finally, the presence of the magnetic field also affects the radiation beam generation (8, 9) and the dose deposition (10, 11).

Magnetic field influence on dose deposition is dependent on the radiation beam orientation relative to the magnetic field and on its strength. In brief, the trajectories of both the contaminant electrons as well as of the secondary electrons are altered by the Lorentz force. In transverse MRI-linacs, this causes the electron paths between collisions to become curved and results in: (1) shifted and asymmetric beam penumbra (10), (2) decreased build-up distance (10), (3) skin dose reduction within and possible increase outside the primary beam (12, 13), and (4) localized dose increase at high-to-low density interfaces due to the electron-return effect (ERE) (14). Inline MRI-linacs instead minimize or even exploit some of these effects. The Lorenz force causes the electrons to spiral around the magnetic field direction and successive energy losses in collisions lead to the shrinkage of their helical orbits (11) which results in: (1) reduction of the beam penumbra (11), (2) dose enhancement on the beam central axis (CAX), especially in low density materials (15), (3) reduction in the dose deposition perturbations due to density heterogeneities (11) and focusing of the contaminate electrons around the radiation beam axis (16, 17). In both perpendicular and parallel orientations these effects, unfamiliar in conventional radiation therapy, require characterization during commissioning.

It should be emphasized that, both the dose deposition in matter as well as the response of the dosimeters are affected by the magnetic field. The trajectories of electrons traversing their active volume change, however this change may be different in the materials constituting the detector (e.g., air cavities in ion chambers, silicon wafers in diode detectors etc.) than in the surrounding medium. As a result, the reading of the detector may not represent the dose that would be deposited in the medium in its absence. Furthermore, many detectors are not symmetric; therefore the change in their response is dependent on their orientation in the magnetic field. These effects have been observed for various types of detectors (18–22) and must be considered both in absolute (21, 23, 24) as well as in relative dosimetry (25). Additionally, air gaps present between the dosimeter and the surrounding material have been shown to influence the detector response (26, 27).

The interaction of the radiation beam and magnetic field renders the commissioning of a MRI-linac a custom task requiring adaptation of existing methods and considerate choice of dosimetric equipment. To date no guidelines on this new type of technology are available (7). For a commercial transverse MRI-linac, dosimetric (28), and imaging-oriented (7) commissioning have been described recently. Inline systems, owing to the fundamental difference in their design, have a set of specific properties which have to be addressed. For the Australian MRI-linac, a high field prototype exploring the inline configuration, the imaging performance, also in the presence of the radiation beam, has been investigated previously (29). The aim of this work was 2-fold:

- to demonstrate the dosimetric properties characteristic to an inline configuration; and
- to commission the radiation-related aspects of the Australian MRI-linac for its application in clinical treatments, in particular:
  - to fine-tune the system for optimal characteristics of the radiation beam;
  - to characterize the its dosimetric components and the radiation beam according to international standards for medical linear accelerators; and
  - to acquire base data for beam modeling in the treatment planning system (TPS) and with Monte Carlo (MC) method.

## Materials and Methods

### The Australian MRI-Linac

The Australian MRI-Linac consists of a dedicated open bore 1 T magnet (Agilent, UK) and a linear accelerator Linatron-MP (Varex, USA) with a stand-alone, clinical multi-leaf collimator (MLC) Millennium (Varian Medical Systems, USA). While the design permits radiation beam entry (and patient positioning) in either orientation, the current system employs inline orientation with a fixed horizontal beam and patient entry through the magnets gap. The system is not equipped with secondary collimators (jaws) and the MLC leaves travel in a horizontal (x) direction with no collimator rotation possible. Uniquely, the linac and the MLC are mounted on rails with a docking system, allowing variation of the source-to-isocentre distance (SID) between 3.2 and 1.8 m and enabling measurements at different magnetic field strengths (2, 29).

The Linatron-MP generates flattening filter free (FFF) photon beams at two nominal energies (4 and 6 MV) and the pulse repetition frequency between 50 and 400 for the 4 MV beam and 50 and 200 for the 6 MV beam. For clinical application, only the 6 MV beam and trigger rate 200 will be used and all the measurements reported in this manuscript were performed with these settings. Machine output is calibrated by the vendor to deliver 1 Gy at d_max_ at 1 m distance per monitoring unit (MU) for an open field (as the linac is equipped with primary 30° conical collimator only) in ~0 T magnetic field.

**Figure 1:**
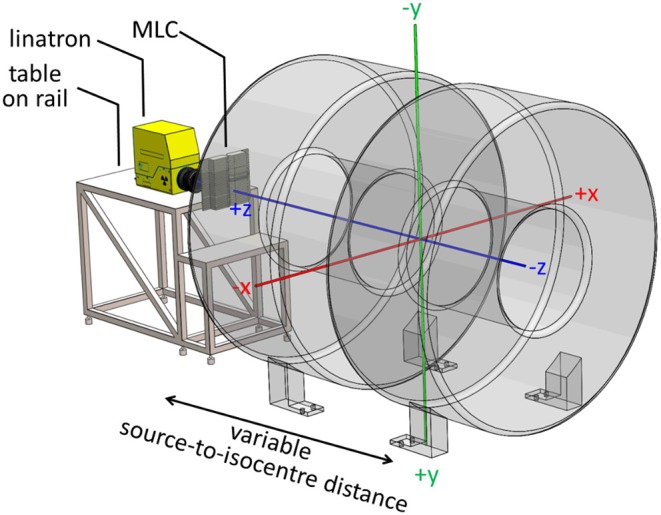
Layout of the Australian MRI-linac with a coordinate system originating at the system's isocentre overlaid.

The conceptual design of the Australian MRI-linac and the coordinate system, originating at the isocentre, used in this work are shown in Figure 1 and further details of the system can be found elsewhere (2, 16).

### Phantoms and Detectors

**Figure 2:**
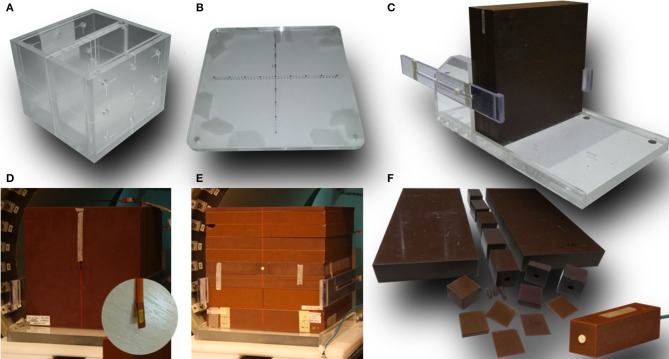
Dedicated phantoms and setups used in this work: **(A)** MRI phantom and **(B)** MV phantom used for system alignment, **(C)** stand for vertical positioning of the solid water slabs, **(D)** setup used for MOSkin™ measurements and a close-up od one of the MOSkin™ detectors, **(E)** setup used for microDiamond measurements, and **(F)** solid water pieces used for the measurements with microDiamond.

For geometrical tests a combination of MRI and MV visible phantoms was used: (1) a dedicated MRI phantom (Leeds Test Objects, UK) consisting two chambers separated by 2 cm thick wall with five narrow bore holes connecting them and filled with MRI visible solution (Figure 2A) and (2) two acrylic plates with embedded fiducial markers for MV visibility (Figure 2B). A stand-alone EPID XRD 1640 AL7-M (PerkinElmer, USA) with a pixel matrix of 1,024 × 1,024 and pixel size 0.4 mm was used for the tests which involved imaging of the measurement setup components using the radiation beam. EPID images were processed using ImageJ software (National Institutes of Health, USA).

For point dose measurements a Farmer-type chamber FC65-G (Scanditronix-Wellhöfer, USA), positioned vertically either in a manual 2D water tank (for absolute does measurements) or in solid water blocks (for relative dose measurements), connected with a bias of 300 V CEP to a Unidos (PTW, Freiburg, Germany) electrometer was used. Whenever the solid water setup was used, the chamber holder was filled with water to avoid the presence of air gaps. The chamber and the electrometer were independently characterized for reproducibility and linearity using a well type strontium source prior to measurements. Finally, the chamber is traceable to the National Physical Laboratory (NPL, UK) and has been calibrated in a 6 MV FFF beam both in 0 T and in 1 T field (30).

For electron contamination characterization and entrance dose measurements a synthetic microDiamond 60019 (PTW, Germany) was used connected to a Unidos electrometer with a bias of 0 V was oriented with the long axis parallel to the beam. The detector's sensitive volume is 2.2 mm in diameter and 1 μm thick and the effective point of measurement (EPOM) is at 1 mm depth and has been determined to be unaffected by the magnetic field (31). While an increased angular dependence for the diamond detector response in a transverse 1.5 T field has been observed (31), which was deemed relevant for relative dosimetry at distant off-axis positions or at different gantry angles, Monte Carlo simulations indicate that this effect is minimized in inline orientation (22). For higher resolution information, these measurements were complemented with the data acquired using MOSkin™ detectors (Figure 2D), developed at Centre for Medical Radiation Physics (CMRP) of the University of Wollongong, which feature an EPOM of 0.07 mm. These detectors were used with their own readout system measuring their gate threshold voltage. As their sensitivity to radiation dose decreases over large voltage ranges, the readout was corrected by taking a reference reading at the beginning and end of each set of measurements. The MOSkin™ detectors have been recently shown to agree with EBT3 films in 1 T inline magnetic field and were deemed suitable for relative dose measurements (32). For both, microDiamond and MOSkin™ measurements customized solid water blocks were used adapted to host the detectors and to enable measurements depth variation (Figures 2D–F).

For beam symmetry and flatness assessment as well as for some profile measurements, the Starcheck maxi MR array (PTW, Germany) was used. The array consists of 707 vented ionization chambers arranged, with 3 mm resolution, along the principal axes and the diagonals of a 40 × 40 cm^2^ area and designed for use in magnetic fields of up to 1.5 T. It was characterized for reproducibility, linearity, sensitivity to misalignment, and geometrical fidelity, based on the IEC60731 (33), both in a 1 T field on the Australian MRI-linac and in a 0 T field on a 6 MV Elekta (Elekta, UK) clinical linear accelerator at the Liverpool Cancer Therapy Centre. Different orientation of individual detectors in detector arrays leads to non-negligible artifacts in profile measurements (34) for transverse MRI-linacs, however these effects have not been observed for the Australian MRI-linac employing an inline configuration. The profiles were analyzed with the Mephysto (PTW, Germany) software accompanying the detector.

Beam depth and cross profiles were acquired using Gafchromic™ EBT3 films (Ashland, USA) placed in solid water blocks, as standard scanning water tanks are not compatible with the MRI-linac systems, due to the presence of metallic components and size restrictions. A dedicated stand (Figure 2C) was constructed to keep the solid water blocks tightly together for the profile measurements in order to eliminate the presence of air gaps. The relative response of the EBT3 films has been shown to be unaffected by the magnetic field (35, 36). The batch of films used was calibrated using a 6 MV beam on an Elekta linear accelerator at the Liverpool Cancer Therapy Centre. The film handling and analysis followed published recommendations (37, 38). The films were scanned using a Perfection V700 Photo (Epson, Japan) flatbed scanner with resolution of 72 dpi and in 48-bit RGB format and all scanner color corrections turned off. A black paper frame was used to position the films at a consistent area of the scanner bed. The orientation of all films was kept constant and aligned to within ±5°. A thin glass plate was placed on top of the films during digitization in order to keep the films flat on the scanner bed. Films were processed and profiles were extracted using ImageJ software (National Institutes of Health, USA).

### System Optimization

#### Magnetic Shielding Optimization

The fringe field affects the radiation beam generation and transport in the linac (8, 9, 39). In particular, (1) it may deflect the electrons produced by the electron gun and reducing the stream of electrons injected to the waveguide and hence the beam output and (2) it may shift the incidence of the electron beam on the target and lead to the deformation of the resulting photon beam profiles and output loss as the beam passes through the primary collimator. Initially, these effects have been reduced by magnetic shielding placed around the target area outside of the x-ray head housing. However, at shorter SIDs (i.e., in higher fringe field) magnetic shielding closer to the target was necessary. To ensure clinically acceptable beam characteristics at the shortest SID, a shield to be placed directly above the beam centerline has been prototyped first, using sheets of μ-metal (Magnetic Shield Corporation, USA), and later manufactured out of iron and fixed to ensure stability and reproducibility. The design of the shield was guided by measurements of the beam output and of the profile symmetry using the Starcheck^maxi−MR^ array. For these measurements, the detector array was placed at the isocentre with build-up material equivalent to 10 cm of water and 10 cm of backscatter material behind it and profiles of varying field sizes were acquired at different SIDs.

#### System Alignment

Alignment of the radiation beam with the MRI scanner imaging isocentre was a two-step process. First, the MRI phantom was scanned using a T1-weighted spin-echo sequence in XZ (resolution: 0.9 × 0.9 × 5 mm) and XY (resolution: 0.8 × 0.8 × 5 mm) planes and realigned iteratively until its position matched the localization of the imaging isocentre. The in-room lasers were then set to indicate the imaging isocentre using the external markings on the phantom. Next, fiducial marker phantoms were added to the setup at the proximal and the distal end of the bore and the whole setup was imaged (at different SIDs) using the EPID placed behind the bore (**Figure 4A**). Based on the projections of the fiducial markers the position of the radiation source was calculated and the linatron was iteratively re-aligned to achieve the best congruence of the radiation isocentre with the imaging isocentre over the range of SIDs. Finally, the half-blocked fields were imaged with EPID placed at two distances: behind the bore and in front of the bore and MLC center position at the isocentre was calculated based on these images to guide the iterative re-alignment of the MLC assembly and fine-tuning of the MLC central axis parameter in the MLC control software.

### Field Size and Leaf Width Calibration

Due to a non-standard distance between the radiation source and the MLC, the magnification factor to apply on the field sizes set in the MLC control software had to be determined. It was obtained by the measurement of the actual field sizes produced by a set of square fields defined in the control software for standard clinical geometry for Millennium MLC, referred to in the reminder of this manuscript as nominal field sizes, using the EPID placed at the distance of 100 cm from the radiation source. The resulting calibration factors were then extrapolated to other SIDs and verified using films placed at the isocentre on the surface of the solid water phantom.

### Functional Performance Characteristics

System characteristics have been benchmarked at commissioned SIDs against the applicable requirements either using or adapting the methods specified in the IEC 60976/977 standard (40, 41). Non-applicable tests included: electron radiation beams, dependencies on angular positions (collimator, gantry), moving beam RT, indicators (light field, front pointer, etc.) not present in the current system and patient support system constituting a separate development.

#### Dose Monitoring System

Reproducibility, proportionality, field size dependence and stability of the dose monitoring system were assessed using the chamber FC65-G placed in a solid water holder at the isocentre with 10 cm build-up and 10 cm backscatter material. 1 Gy irradiations with fields of ~10 × 10 cm^2^ were performed for the reproducibility measurements and with ~20 × 5 and ~5 × 20 cm^2^ fields for the field size dependency measurements. Proportionality was assessed over a range of doses from 0.1 to 10 Gy. Stability after a high absorbed dose was assessed as the difference in measurements prior to and after a 30 min period of irradiation and stability throughout the week was assessed through measurements on 5 subsequent days following 3 h of stand-by mode. All stability tests have been performed at dose of 1 Gy for an ~10 × 10 cm^2^ field. Additionally, the magnitude of the magnetic field at the position of the monitoring chamber was recorded using a VGM gaussmeter (AlphaLab, USA).

#### Depth Dose Characteristics

Percentage depth dose (PDD) curves for ~10 × 10 cm^2^ and maximum field sizes were acquired using EBT3 films placed in solid water blocks and aligned parallel to the beam axis at SSD = SID−10 cm. PDDs were extracted along the beam central axis and at ±3.5 cm off-axis in order to assess the depth of dose maximum (d_max_) and the penetrative quality, defined as depth at which the dose amounts to 80% of the maximum dose (D_max_) (40, 41). Additionally, films placed at the surface of the phantom perpendicular to the beam direction were used for surface dose measurements.

Increased dose in the initial few centimeters around the beam central axis due to the contaminant electron focusing in the fringe field of the magnet has been previously both, modeled (11, 42) and observed experimentally on an earlier (16) and the current prototype (32). To characterize this effect in detail for the current system and to explore possible methods to mitigate it (off-axis irradiation, use of bolus), microDiamond and MOSkin™ detectors were used. The detectors were placed in solid water blocks adapted to enable data acquisition at variable depths or covered with increasing layers of kapton tape.

Beam quality was characterized using tissue phantom ratio (TPR_20/10_) measured using the FC65-G ion chamber in solid water using a field closest to 10 × 10 cm^2^ and the dose of 1 Gy.

#### Beam Uniformity

Beam symmetry and flatness were measured using the Starcheck^maxiMR^ array for fields closest to 5 × 5, 10 × 10, 30 × 30 cm^2^, and the maximum field size at commissioned SIDs. The detector array was placed at the isocentre with water equivalent material amounting to 10 cm of build-up and 10 cm of back scatter. Beam symmetry was determined as the maximum ratio of the doses at any two positions symmetrical to the beam axis and flatness as the ratio between the maximum and the minimum dose inside the flattened area (40, 41).

Beam penumbra (20–80%) was measured at the isocentre at 10 cm depth. For fields of ~5 × 5 and 10 × 10 cm^2^ it was extracted from EBT3 films placed perpendicular to the beam axis within solid water blocks. For the 30 × 30 cm^2^ and the maximum commissioned field sizes, which exceed the size of the solid water blocks, penumbra measured with the Starcheck^maxiMR^ array and dedicated build-up plates is reported. In order to apply the standard 20–80% definition for penumbra evaluation, only the field edge sections of the FFF profile have to be considered (43). This was achieved by identifying the profile inflection points using a method which calculates the third derivative of the profile, proposed by Fogliata et al. (44), and renormalizing profiles to these points.

#### Isocentre

Congruence of the imaging and radiation isocentre, expressed as horizontal (x) and vertical (y) offset between the beam focal position and the in-room lasers, was measured using the setup and methods described in section System Alignment.

#### Geometry of the Beam Limiting System

Symmetry of the opening around the imaging isocentre and parallelism of the leaves to the in-room lasers have been tested here, since the current system is not equipped with diaphragms. These tests have been performed using the setup and methods described in section System Alignment. The latter was measured as the angle between the line defined by the projection of the fiducials and projection of the MLC CAX in EPID images (see **Figure 6**) using ImageJ software.

### Base Data Acquisition

Beam characterization has been performed at commissioned SIDs and SSD = SID−10 cm following the AAPM Task Group 106 recommendations (45). Non-applicable procedures included elements not available in the current system: measurements of tray and wedge factors, tests of the light field and radiation field concurrence and characterization of the electron beams. In the initial clinical phase, the MRI-linac will be used only for static 3D conformal treatments, hence only the acquisition of the input data relevant for such treatments is reported in this work.

#### Depth Dose Characteristics and Surface Dose

PDDs were measured for square fields of up to ~18 × 18 cm^2^ and two rectangular fields of ~18 × 6 and ~6 × 18 cm^2^ using EBT3 films placed in solid water blocks and aligned parallel to the beam axis. Additionally, films placed at the surface of the phantom perpendicular to the beam direction were used for surface dose measurements. PDDs were extracted along the central axis.

#### Beam Profiles

Beam profiles were acquired for square fields of up to ~18 × 18 cm^2^ and two rectangular fields of ~18 × 6 and ~6 × 18 cm^2^ at depths of 1, 5, 10, and 20 cm. EBT3 films were placed perpendicular to the beam direction in solid water blocks.

#### Tissue Phantom Ratios

Beam quality measurements as required by IEC 60976/977 standard (40, 41) were addressed as part of functional performance characterization in section Depth Dose Characteristics.

#### Output Factors

Total scatter factors were measured in solid water using the FC65-G ion chamber and the microDiamond detector for field sizes from the smallest available to ~25 × 25 cm^2^. Collimator scatter factors were measured at 10 cm depth using the FC65-G ion chamber in a GEC-ESTRO mini phantom placed horizontally (46). Results were normalized to the field closest to 10 × 10 cm^2^.

#### Beam Output Calibration

Absolute dosimetry was performed following the TRS-398 protocol (47). Output was measured in a manual 2D water tank in 10 cm depth under isocentric conditions for square fields closest to 10 × 10 cm^2^ using FC65-G ion chamber calibrated in the magnetic field and traceable to NPL as described above. Corrections for polarity, recombination, ambient room conditions and magnetic field were applied. Polarity was measured via acquisition of output with opposite polarizing potentials (−300 and +300 V) applied to the chamber and yielded k_pol_ = 1.0005. Recombination was measured via the two voltage method using polarizing potentials of −300 and −100 V and yielded k_s_ = 1.0015. Calibration in 1 T yielded a k_B_ factor of 0.99 (30). Correction of k_FFF_ = 1.003 was used for FFF beam volume averaging effects. A CC13 chamber was used to normalize the output between measurements.

#### MLC Characterization

Positional accuracy of the MLC (symmetry around the isocentre and tilt) has been assessed as described in section Geometry of the Beam Limiting System and beam penumbra was measured with EBT3 films as described in section Beam Uniformity.

The MLC transmission measurements were performed using a method similar to one described by Arnfield et al. (48) and Patel et al. (49) with leaves fully closed and the gap between the opposing leaf pairs displaced to one side. Average MLC transmission was measured using the FC65-G ion chamber placed in solid water perpendicular to the leaf travel direction at the isocentre at depth of 11 cm. Simultaneously, the intra- and interleaf leakage was measured using an EBT3 film placed at 10 cm depth.

## Results

### System Optimization

#### Magnetic Shielding Optimization

**Figure 3:**
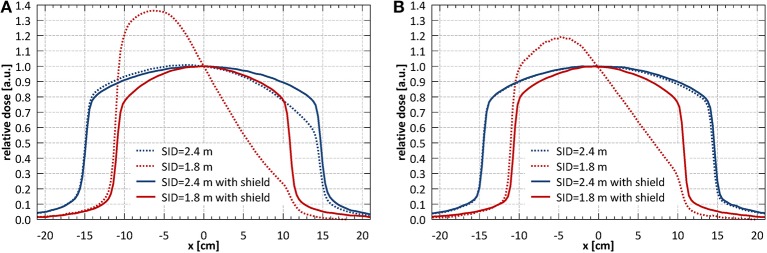
**(A)** Horizontal (x) and **(B)** vertical (y) profiles of a nominal 11 × 11 cm^2^ field acquired at different SIDs with only external magnetic shielding (dotted lines) and with optimized internal magnetic shielding (solid lines).

Figure 3 shows the profiles of a nominal 11 × 11 cm^2^ field acquired at different SIDs with only external magnetic shielding (dotted lines) and with optimized internal magnetic shielding (solid lines). The shielding significantly improved the profile symmetry even at shortest SID and reduced the beam output loss in the target area. It should be noted however that, located relatively far from the electron gun, it was not effective in reducing the beam loss occurring there. Based on these results the system has been commissioned at two SIDs: at 2.4 m, where full range of fields (up to 34.3 × 34.0 cm^2^ at the isocentre) fulfilled the symmetry criteria, and at 1.8 m, where fields of up to 21.4 × 21.2 cm^2^ fulfilled the symmetry criteria. The measurements reported in the reminder of this manuscript have been performed at these two SIDs, unless stated otherwise.

#### System Alignment

**Figure 4:**
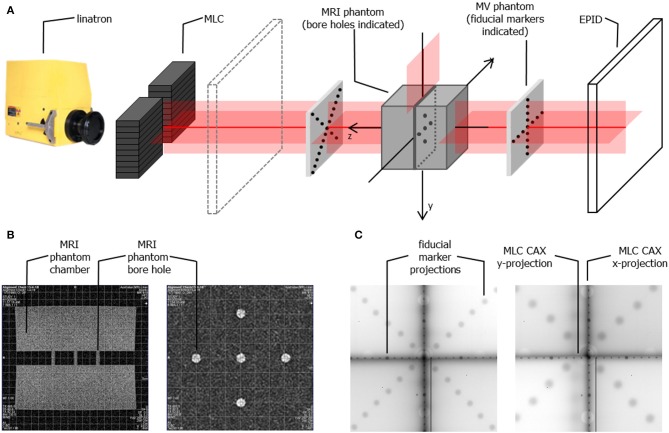
**(A)** Schematic representation of the phantom setup used for geometrical alignment of the system **(B)** MR scans of the alignment phantom in x-z plane (left) and x-y plane (right) showing the bore holes aligned to the imaging isocentre (indicated by the superimposed grid) **(C)** example composite portal images showing the projections of the fiducial markers (aligned to the in-room lasers) and of the edges of half blocked fields formed by the MLC for SID of 2.4 m (left) and SID of 1.8 m (right). Composite images are created as: |image_negativexblocked_ – image_positivexblocked_| + |image_negativeyblocked_ – image_positiveyblocked_| allowing visualization of the MLC axes.

The MRI scans of the alignment phantom with the grid indicating the imaging isocentre superimposed are shown in Figure 4B and the measured agreement was better than 1 mm.

The physical change in position of the linatron relative to the origin axis over the rail length was 2 mm horizontally (x) and 3 mm vertically (y).

**Figure 5:**
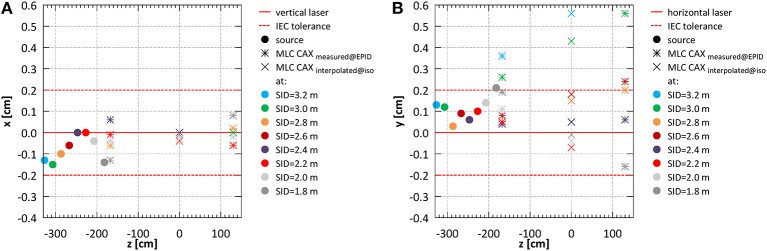
System alignment for all SIDs **(A)** in the horizontal (x) and **(B)** in the vertical (y) direction.

Example composite portal images, showing the projections of the fiducial markers (as indicators of the laser positions) and of the edges of half blocked fields formed by the MLC, are shown in Figure 4C. Composite images were created as: |image _negativexblocked_ – image_positivexblocked_| + |image_negativeyblocked_ – image_positiveyblocked_| allowing visualization of the MLC axes. Alignment of radiation isocentre and the MLC center relative to the positioning lasers for different SIDs is summarized in Figure 5. The results indicate a variation of the radiation focal spot offset (circles in Figure 5) from to the laser with changing SID of up to 1.5 ± 2.5 mm in the horizontal (x) direction and 1.4 ± 1.8 mm in the vertical (y) direction, except at SID of 1.8 m where it reached 2.1 ± 1.6 mm. This leads to the variation of the position of the MLC CAX projection with changing SID, which in the horizontal (x) direction could be compensated for using an SID specific parameter setting in the MLC control software.

Offsets of the MLC CAX projection position with respect to the lasers measured in EPID images acquired in front of the magnet bore (*z* = −167.6 cm) and behind the magnet bore (*z* = 130.1 cm) (bars in Figure 5) were used to interpolate the MLC CAX projection at the isocentre (*z* = 0 cm) (×'s in Figure 5). In the horizontal (x) direction it was within 0.4 ± 1.9 mm at all SIDs. In the vertical (y) direction it was within 1.8 ± 2.2 mm at all SIDs except the largest two (3.2 and 3.0 m).

### Field Size and Leaf Width Calibration

The measured field sizes were 7.2% larger in horizontal (x) and 6.1% larger in vertical (y) direction than the nominal field sizes. Extrapolated to the two commissioned SIDs this yielded magnification factors of 2.638 and 2.612 in horizontal and vertical direction for SID of 2.4 m and 1.944 and 1.924 in horizontal and vertical direction for SID of 1.8 m. This was incorporated in the TPS in the definition of the MLC leaf projection widths in the isocentre plane.

The full width half maximum of the surface profiles acquired using EBT3 film for fields closest to 10 × 10 cm^2^: 10.6 × 10.5 cm^2^ at SID of 2.4 m and 9.7 × 9.6 cm^2^ at SID of 1.8 m were 10.6 × 10.1 and 9.7 × 9.6 cm^2^ respectively.

### Functional Performance Characteristics

#### Dose Monitoring System

**Figure 6:**
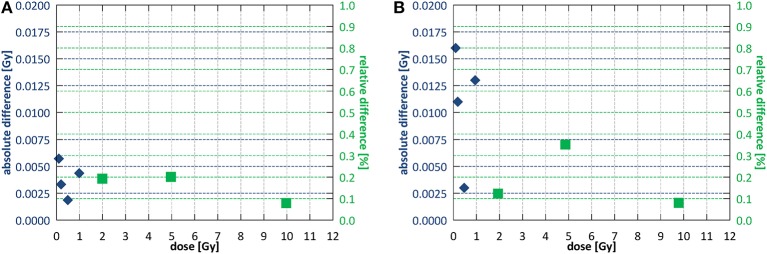
Dose output linearity **(A)** at SID of 2.4 m and **(B)** at SID of 1.8 m. Different symbols indicate regions of applicability of the absolute and the relative deviation criterion according to IEC60976 (39).

For reproducibility, proportionality and stability measurements fields of 10.6 × 10.5 and 9.7 × 9.6 cm^2^ were used for SID of 2.4 and 1.8 m, respectively. Short term reproducibility of the monitoring chamber calculated as a coefficient of variation (40) was 0.29% at SID of 2.4 m and 0.49% at SID of 1.8 m. Output after high absorbed dose showed a decrease in of 1.2 ± 0.4% at SID of 2.4 m and 1.1 ± 0.4% at SID of 1.8 m. Stability throughout the week was 1.0 ± 0.6% at SID of 2.4 m and 2.6 ± 0.6% at SID of 1.8 m. Stability after a full day of intensive commissioning measurements yielded 1.7 ± 0.4 and 2.8 ± 1.0% output decrease at SID of 2.4 and 1.8 m, respectively, however such intensive clinical use of the system is not foreseen outside of commissioning or annual quality assurance. Figure 6 shows the dose output linearity, calculated as per IEC60977 (41), for both commissioned SIDs. At SID of 2.4 m, linearity was better than 0.2% above 1 Gy and better than 0.006 Gy below 1 Gy. At SID of 1.8 m, linearity was better than 0.4% above 1 Gy and better than 0.016 Gy below 1 Gy.

For the measurements of the dependence on the field shape, fields of 5.3 × 20.9 and 21.1 × 5.2 and 5.8 × 19.2 and 19.4 × 5.8 cm^2^ were used for SID of 2.4 and 1.8 m, respectively. Variation with the field size shape was 1.3 ± 0.4% at SID of 2.4 m and 0.0 ± 0.5% at SID of 1.8 m.

The fringe field magnitude at the location of the monitoring chamber was ~15 and 45 mT for SID of 2.4 and 1.8 m, respectively.

#### Depth Dose Characteristics

**Figure 7:**
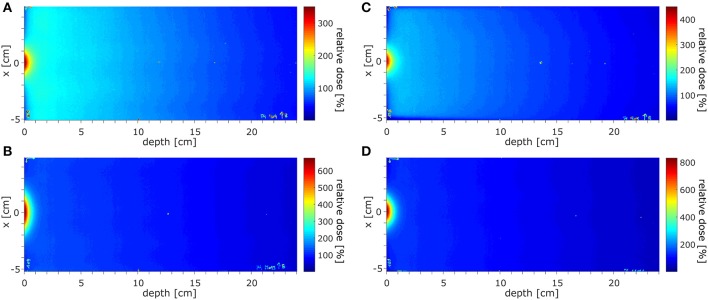
The dose distributions acquired in the region of ±10 cm around the beam CAX, normalized at depth of 10 cm, **(A,B)** at SID of 2.4 m for field sizes of 10.6 × 10.5 and 34.3 × 34.0 cm^2^ and **(C,D)** at SID of 1.8 m for field sizes 9.7 × 9.6 and 21.4 × 21.2 cm^2^.

The dose distributions acquired using EBT3 films in the region of ~±10 cm around the beam CAX, normalized at depth of 10 cm, at SID of 2.4 m for field sizes of 10.6 × 10.5 and 34.3 × 34.0 cm^2^ are shown in Figures 7A,B and at SID of 1.8 m for field sizes of 9.7 × 9.6 and 21.4 × 21.2 cm^2^ in Figures 7C,D. The higher dose around the central axis at small depths caused by electron focusing is visible.

The surface doses (normalized to 10 cm depth) measured using EBT3 films were: 430% for 10.6 × 10.5 cm^2^ field and 1,024% for 34.3 × 34.0 cm^2^ at SID of 2.4 m and 576% for 9.7 × 9.6 cm^2^ field and 1,068% for 21.4 × 21.2 cm^2^ at SID of 1.8 m. Measured ±3.5 cm off-axis, at SID of 2.4 m these values were reduced to 79% for 10.6 × 10.5 cm^2^ field and 143% for 34.3 × 34.0 cm^2^ and at SID of 1.8 m to 72% for 9.7 × 9.6 cm^2^ field and 103% for 21.4 × 21.2 cm^2^. The radius at which the surface dose becomes lower than the dose at 10 cm depth was between 2.6 cm for 10.6 × 10.5 cm^2^ field and more than 6 cm for 34.3 × 34.0 cm^2^ field at SID of 2.4 m and between 2.6 cm for 9.7 × 9.6 cm^2^ field and 4 cm for 21.4 × 21.2 cm^2^ at SID of 1.8 m.

High dose deposited in the initial section of the PDD by the contaminant electrons focused around the beam central axis hinders the determination of the depth of d_max_ and of the penetrative quality of the beam in the radiation field according to the IEC 60976/977 standard (40, 41). As estimates, these values were extracted ±3.5 cm off-axis yielding: 1.47 cm for field 10.6 × 10.5 cm^2^ and 1.54 cm for field 34.3 × 34.0 cm^2^ at SID of 2.4 m and 1.45 cm for field 9.7 × 9.6 cm^2^ and 1.49 cm for field 21.4 × 21.2 cm^2^ at SID of 1.8 m. The penetrative quality was 7.19 cm for the 10.6 × 10.5 cm^2^ at SID of 2.4 m and 7.02 cm for the 9.7 × 9.6 cm^2^ at SID of 1.8 m.

**Figure 8:**
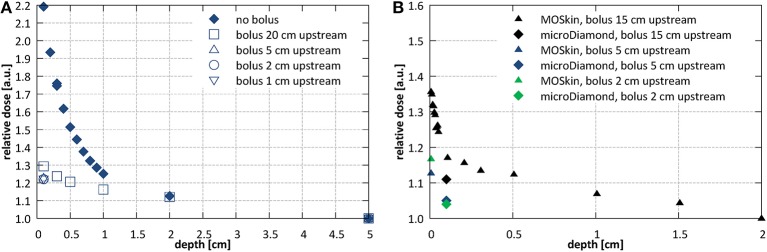
Investigations of the entrance dose at SID of 2.4 m with varying bolus placement **(A)** with microDiamond for a 10.6 × 10.5 cm^2^ field at SID of 2.4 m (normalized at depth of 5 cm) and **(B)** with MOSkin™ and microDiamond detector for a 7.9 × 7.8 cm^2^ field (normalized at depth of 2 cm).

Use of a bolus placed upstream of the entrance surface to mitigate the presence of the contaminant electrons was investigated. Bolus thickness of 2 cm was selected based on the observed penetration depth of the electrons. Figure 8A shows the initial section of the PDD acquired using the microDiamond detector for a 10.6 × 10.5 cm^2^ field at SID of 2.4 m with bolus placed 20, 10, 5, 2, and 1 cm upstream from the surface of the phantom. Measurements were normalized to the dose at 5 cm depth due to the contaminant electrons affecting the dose maximum position. The presence of the bolus lead to a significant reduction of the electron hotspot: from more than 220% at 1 mm depth to about 120–130%, depending on the distance at which the bolus was placed. Placing the bolus close to the surface, i.e., reducing the length of the air column where new electrons can be generated, resulted with lower surface dose, however only down to a distance of ~5 cm upstream from the phantom surface. Figure 8B shows higher resolution data (normalized at depth of 2 cm) acquired with the MOSkin™ detector, which reveal presence of a further dose enhancement and steep dose fall-off within the initial 1 mm of the PDD, i.e., at depths smaller than the EPOM of the microDiamond.

For TPR_20/10_ measurements fields of 10.6 × 10.5 and 9.7 × 9.6 cm^2^ were used for SID of 2.4 and 1.8 m, respectively. The measured TPR_20/10_ values were 0.633 ± 0.001 at SID of 2.4 m and 0.634 ± 0.004 at SID of 1.8 m.

#### Beam Uniformity

Results of the beam symmetry and flatness measurements performed with the Starcheck^maxiMR^ array are summarized in Table 1 for SID = 2.4 m and in Table 2 for SID of 1.8 m. At the larger SID, the IEC criteria for symmetry were fulfilled for fields up to 34.3 × 34.0 cm^2^ while at shorter SID for fields up to 21.4 × 21.2 cm^2^.

**Table 1:** Beam symmetry, flatness and penumbra at depth of 10 cm at SID of 2.4 m.

**Field size (cm^2^)**	**Symmetry (%)**		**Flatness**		**Penumbra (cm)**	
	**x**	**y**	**x**	**y**	**x**	**y**
5.3 × 5.2	100.4	100.3	1.04	1.03	1.06^+^ 0.90^*^	0.66^+^ 0.73^*^
10.6 × 10.5	100.4	100.5	1.08	1.08	1.35^+^ 1.10^*^	0.86^+^ 0.84^*^
29.0 × 28.7	101.8	100.6	1.17	1.15	n.a.^+^ 1.48^*^	n.a.^+^ 1.29^*^
34.3 × 34.0	102.2	100.4	1.22	1.22	n.a. ^+^ 1.49^*^	n.a. ^+^ 1.23^*^

* *Measured with Starcheck ^maxiMR^*.

+ *Measured with film*.

**Table 2:** Beam symmetry, flatness and penumbra at depth of 10 cm at SID of 1.8 m.

**Field size (cm^**2**^)**	**Symmetry (%)**		**Flatness**		**Penumbra (cm)**	
	**x**	**y**	**x**	**y**	**x**	**y**
5.8 × 5.8	101.4	100.8	1.05	1.04	0.89^+^ 0.86^*^	0.56^+^ 0.67^*^
9.7 × 9.6	102.0	101.6	1.10	1.09	1.02^+^ 0.94^*^	0.74^+^ 0.76^*^
21.4 × 21.2	101.5	102.9	1.18	1.18	n.a.^+^ 1.17^*^	n.a.^+^ 1.01^*^

* *Measured with Starcheck ^maxiMR^*.

+ *Measured with film*.

Beam penumbra measured with films (for field sizes not exceeding the size of the solid water blocks) and with the Starcheck array (for all investigated field sizes) at 10 cm depth is summarized in Table 1 for SID of 2.4 m and in Table 2 for SID of 1.8 m. The results obtained with films and with the array agree on average within 1 mm. In the direction perpendicular to the leaf motion (y), where the penumbra is steeper, compared to the direction perpendicular to the leaf motion (y), the penumbra values were 3 mm lower when measured with films and 4 mm lower when measured with the array.

#### Isocentre

Offset of the radiation focal spot from the lasers defining the position of the imaging isocentre was 0.0 ± 2.1 mm in the horizontal (x) and 0.6 ± 2.1 mm in the vertical (y) direction at SID of 2.4 m and −1.4 ± 1.6 mm in the horizontal and 2.1 ± 1.6 mm in the vertical direction at SID of 1.8 m.

#### Geometry of the Beam Limiting System

Offset of the projection of the MLC center from the lasers defining the position of the imaging isocentre was 0.0 ± 2.1 mm in the horizontal (x) and 0.8 ± 2.1 mm in the vertical (y) direction at SID of 2.4 m and −0.1 ± 1.6 mm in the horizontal and −0.2 ± 1.6 mm in the vertical direction at SID of 1.8 m. The MLC bank tilt was 0.28° measured at SID of 2.4 m and 0.13° measured at SID of 1.8 m.

### Beam Base Data Acquisition

Based on the surface dose measurements performed as part of the functional characteristic tests, the base data relevant for beam modeling (PDDs, beam profiles and absolute dose calibration) was acquired for both cases: without and with the electron absorbing bolus placed 5 cm upstream from the surface of the phantom, as currently its use is foreseen for first patient treatments.

#### Depth Dose Characteristics and Surface Dose

**Figure 9:**
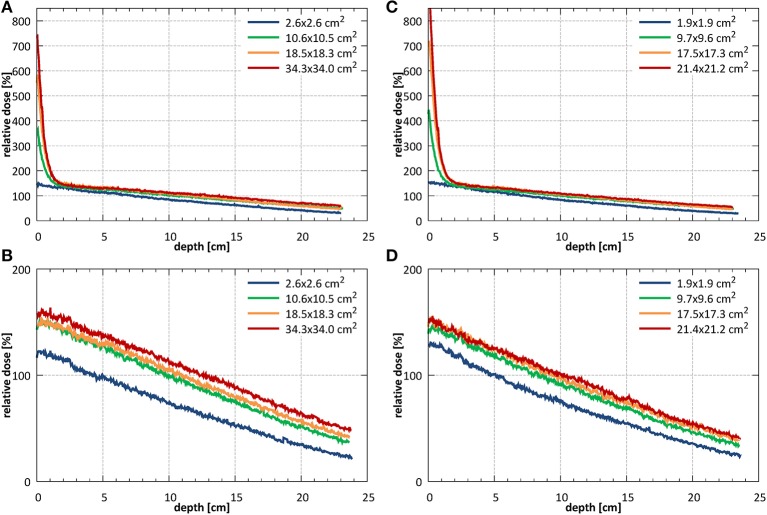
PDDs measured **(A)** without **(B)** with the bolus at SID of 2.4 m for field sizes 2.6 × 2.6, 10.6 × 10.5, 18.5 × 18.3, and 34.3 × 34.0 cm^2^ (normalized to the 10.6 × 10.5 cm^2^ field at a depth of 10 cm) and **(C)** without and **(D)** with the bolus at SID of 1.8 m for field sizes 1.9 × 1.9, 9.7 × 9.6, 17.5 × 17.3, and 21.4 × 21.2 cm^2^ (normalized to the 9.7 × 9.6 cm^2^ field at a depth of 10 cm).

Example PDD curves measured at SID of 2.4 m for field sizes 2.6 × 2.6, 10.6 × 10.5, 18.5 × 18.3, and 34.3 × 34.0 cm^2^ normalized to the 10.6 × 10.5 cm^2^ field at a depth of 10 cm are shown in Figure 9A without and Figure 9B with the bolus. Example PDD curves measured at SID of 1.8 m for field sizes 1.9 × 1.9, 9.7 × 9.6, 17.5 × 17.3, and 21.4 × 21.4 cm^2^ normalized to the 9.7 × 9.6 cm^2^ field at depth of 10 cm are shown in Figure 9C without and Figure 9D with the bolus.

#### Beam Profiles

**Figure 10:**
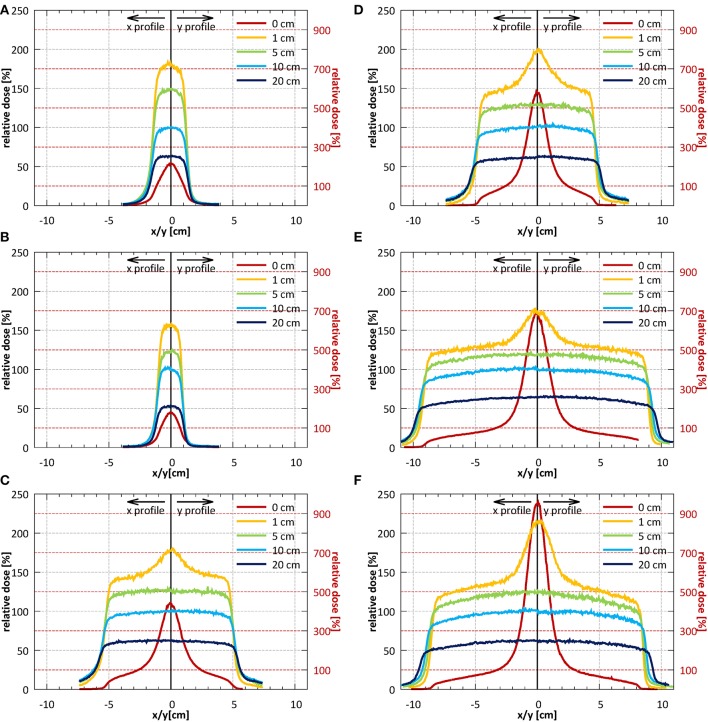
Profiles in the horizontal (x) direction (solid lines) and in the vertical (y) direction (dotted lines) measured without the bolus at the surface and at depths of 1, 5, 10, and 20 cm **(A–C)** at SID of 2.4 m for field sizes 2.6 × 2.6, 10.6 × 10.5, and 18.5 × 18.3 cm^2^ (normalized to CAX value of the 10.6 × 10.5 cm^2^ field at a depth of 10 cm) and **(D–F)** at SID of 1.8 m for field sizes 1.9 × 1.9, 9.7 × 9.6, and 17.5 × 17.3 cm^2^ (normalized to CAX value of the 9.7 × 9.6 cm^2^ field at a depth of 10 cm). Note: the secondary y-axis was used for surface profiles and primary y-axis was used for all remaining profiles.

Example profiles acquired at depths of 1, 5, 10, and 20 cm at SID of 2.4 m for field sizes 2.6 × 2.6, 10.6 × 10.5, and 18.5 × 18.3 cm^2^ normalized to the CAX value of the profile of the 10.6 × 10.5 cm^2^ field at 10 cm depth are shown in Figures 10A–C. Example profiles acquired at SID of 1.8 m for field sizes 1.9 × 1.9, 9.7 × 9.6, and 17.5 × 17.3 cm^2^ normalized to the CAX value of the profile of the 9.7 × 9.6 cm^2^ field at 10 cm depth are shown in Figures 10D–F. Horizontal (x) half-profiles are shown on the negative and corresponding vertical (y) half-profiles on the positive x axis. Only data measured without the bolus is shown.

#### Tissue Phantom Ratios

Results of the beam quality measurements required by IEC 60976/977 standard (40, 41) are presented as part of functional performance characterization in section Depth Dose Characteristics.

#### Output Factors

**Figure 11:**
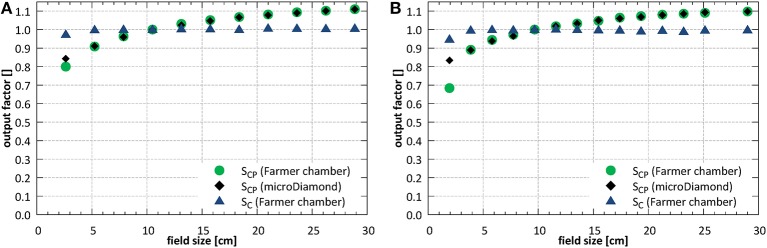
Total (S_CP_) and collimator (S_C_) scatter factors **(A)** at SID of 2.4 m and **(B)** at SID of 1.8 m.

Field size output factors were measured for fields between 2.6 × 2.6 and 26.4 × 26.1 cm^2^ at SID of 2.4 m and normalized to the field of 10.6 × 10.5 cm^2^. At SID of 1.8 m, the field sizes between 1.9 × 1.9 and 25.3 × 25.0 cm^2^ were used and the results were normalized to the field of 9.7 × 9.6 cm^2^. The total (S_CP_) and collimator (S_C_) scatter factors measured under isocentric conditions are shown in Figure 11.

At SID of 2.4 m the total scatter factors measured with the ion chamber and with the microDiamond detector showed a very good agreement for field sizes down to ~5 × 5 cm^2^, with an average deviation of 0.3%. Below, at field size of 2.6 × 2.6 cm^2^, the ion chamber underestimated the output by 5.1%. At SID of 1.8 m the agreement between ion chamber and microDiamond was good down to field size of ~4 × 4 cm^2^, with an average deviation of 0.2%. Below, at field size of 1.9 × 1.9 cm^2^, the ion chamber underestimated the output by 17.9%.

#### Beam Output Calibration

Beam output calibration was performed at a field size of 10.6 × 10.5 and 9.7 × 9.6 cm^2^ for SID of 2.4 and 1.8 m, respectively. The measured beam output was 0.1376 ± 0.0002 Gy/MU at SID of 2.4 m and 0.0915 ± 0.0002 Gy/MU at SID of 1.8 m. The respective factors measured with the electron absorbing bolus in place were 0.1247 ± 0.0001 Gy/MU at SID of 2.4 m and 0.0830 ± 0.0001 Gy/MU i.e., 9.42 and 9.37% lower.

#### MLC Characterization

Positional accuracy of the MLC (symmetry around the isocentre and tilt) is presented in section Geometry of the Beam Limiting System and the measured beam penumbra values in section Beam Uniformity.

**Figure 12:**
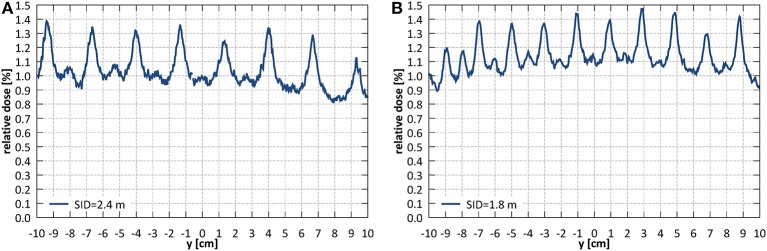
Leakage dose (relative to an open field dose) through a closed MLC **(A)** at SID of 2.4 m and **(B)** at SID of 1.8 m.

Average transmission through the leaves, relative to an open field dose, measured with the EBT3 film (averaged within a radius of 2.5 cm around the center of the field) at SID of 2.4 m was 1.06% and at SID of 1.8 m 1.17%. The corresponding ion chamber measurements were slightly higher and yielded 1.23 and 1.52%, respectively. Interleaf transmission peak-to-peak amplitude was ~0.4% at SID of 2.4 m and 0.3% at SID of 1.8 m (Figure 12).

## Discussion

This work is the first report on the dosimetric characterization of an inline MRI-linac system.

The Australian MRI-linac features a rail system which enables variation of the source-to-isocentre distance. The largest distance (3.2 m) corresponds to the decoupling of the MRI and linac components (B ≈ 0 T). On the other hand, the fringe field at the linac location for the shortest distance (1.8 m) influences the electron transport, and therefore radiation beam generation in the linac, leading to output loss and profile distortion. In order to utilize the shortest SIDs, physical alignment of the radiation beam to the magnetic field was fine-tuned and magnetic shielding of the radiation head was optimized. Aligning and shielding allowed the system commissioning at two SIDs: at 2.4 m where full range of field sizes fulfilled the symmetry criteria and at 1.8 m where fields up to ~21 × 21 cm^2^ fulfilled the symmetry criteria, but the clinical treatments will benefit from smaller leaf width projection and sharper penumbra.

Alignment of the radiation field and the imaging isocentre below the 2 mm tolerance specified in IEC standard was obtained: the deviation of the center of MLC shaped fields from the isocentre was 0.0 ± 2.1 mm in the horizontal (x) and 0.8 ± 2.1 mm in the vertical (y) direction at SID of 2.4 m and −0.1 ± 1.6 mm in the horizontal (x) and −0.2 ± 1.6 mm in the vertical (y) direction at SID of 1.8 m. These offsets stem from the limitations of physical linac and MLC assembly alignment and vary with the SID due to factors such as: residual angle between the rail system, the linac and the magnetic field axis as well as the influence of the fringe field on the electron beam in the linac and hence on its point of incidence on the target. While in the horizontal (x) direction this could be minimized by software-controlled adjustment of the MLC axis, in the vertical direction (y) a compromise in the MLC height placement was made. MLC leakage yielded 1.06% at SID of 2.4 m and 1.17% at SID of 1.8 m measured with film and 1.23% at SID of 2.4 m and 1.52% at SID of 1.8 m when measured with an ionization chamber. In similar measurement, Arnfield et al. (48) reported a value of 1.34 ± 0.03% for the same MLC model.

Profile symmetry was better than 103% for the commissioned field size ranges and the flatness values of 1.03–1.22 were comparable with values reported for a 6 MV FFF beam in literature (50). Penumbra values could be measured with films, offering high spatial resolution, only for a subset of fields required by IEC standard. For an ~10 × 10 cm^2^ field the penumbra was 1.35 cm in the leaf motion direction (x) and 0.86 cm in the direction perpendicular to the leaf motion (y) at SID of 2.4 m and 1.02 cm in the leaf motion direction and 0.74 cm in the direction perpendicular to the leaf motion at SID of 1.8 m. For comparison, penumbra values measured for the same field size on a commercial transverse 1.5 T system, equipped with an Elekta Agility MLC, with an SID of 1.4 m determined using artificial flattening were between 0.74 and 0.87 cm (28). No penumbra asymmetry and profile shift, characteristic for transverse systems (28), has been observed, which simplifies beam modeling in the TPS for inline systems.

The dose monitoring system of the Linatron-MP consists of a single, parallel plate, unsealed monitoring chamber. Reproducibility and proportionality of the chamber met the IEC criteria. However, at shorter SID, the long-term stability over 1 week reached 2.6 ± 0.6%, exceeding the IEC defined tolerance. An independent monitoring chamber is currently being installed to mitigate this for patient treatments as well as to ensure dose monitoring redundancy as per IEC requirements (40, 41). Fringe field effects on beam the beam properties (e.g., presence of the focused electrons, backscatter) and on the dose monitoring system response required a separate beam output calibration at the two commissioned SIDs. Beam quality instead remained the same at both distances (TPR_20,10_ was 0.633 ± 0.001 at SID of 2.4 m and 0.634 ± 0.004 at SID of 1.8 m) within measurement uncertainty. It should be noted that in inline configurations, similar to transverse systems, TPR_20,10_ as opposed to %dd(10)_x_ is more applicable as beam quality measure. Although, contrarily to transverse systems, photon build-up remains unaffected by the inline magnetic fields, determination of d_max_ and D_max_ may be confounded by the presence of electron focusing. In this work, as an approximate estimate the value of d_max_ measured off-axis was reported and amounted to 1.47 cm at SID of 2.4 m and 1.45 cm at SID of 1.8 m for ~10 × 10 cm^2^ fields. For comparison, a reduced d_max_ of 1.3 cm was reported for a 7 MV FFF beam of a commercial 1.5 T transverse system (28).

Electron focusing effect, modeled (42) and observed experimentally (16, 32), was quantified and methods to minimize it have been investigated. Surface dose enhancement around the central axis was observed, which was dependent on the field size and reached 400–600% for 10 × 10 cm^2^ fields and more than 1,000% for largest fields, relative to the dose at 10 cm depth. This could be counteracted by placing of an absorbing bolus upstream of the phantom or by irradiation using off-axis fields. The former resulted in significant reduction of the entrance dose although keeping the maximum dose value on the surface: ~140–150% for ~10 × 10 cm^2^ fields and 160% for small fields, relative to the dose at 10 cm depth. The efficacy of the latter is dependent on the off-axis distance: the distance at which the surface dose becomes lower than the dose in 10 cm depth was between 2.6 cm for 10.6 × 10.5 cm^2^ field and more than 6 cm for 34.3 × 34.0 cm^2^ field at SID of 2.4 m and between 2.6 cm for 9.7 × 9.6 cm^2^ field and 4 cm for 21.4 × 21.2 cm^2^ at SID of 1.8 m.

For absolute dose measurements, the correction factor k_B_ was applied to the ionization chamber reading. However, it should be emphasized, that the effect of the magnetic field on the dosimeters and the sensitivity to the detector orientation has been shown to be less pronounced in inline as compared to transverse configuration (19, 20). To avoid the effect of air gaps, the chamber holder has been filled out with water whenever applicable in this work. Nevertheless, it should also be noted that these effects have been shown to be smaller for the inline relative to the transverse configuration: 0.4% (30) compared to 0.7–1.2% (26).

The beam base data acquired with the bolus has been inserted into Pinnacle (Philips Healthcare, The Netherlands) TPS system for beam modeling, as currently its use is foreseen for first patient treatments. This data as well as data acquired without the bolus will be instead used to improve and validate the MC model of the system.

Last but not least, while this work focuses of strictly dosimetric aspects of the Australian MRI-linac, its imaging performance, including potential interactions between imaging and beam delivery, had been described previously (29). It should also be emphasized that the integration of the whole system has been tested and that the first live animal treatments have been conducted recently (51), as a further step prior to clinical treatments.

## Conclusion

Owing to the fundamentally different design, the inline systems display a different set of dosimetric issues as compared to transverse designs, most notably: no field shift and penumbra asymmetry, no build-up depth reduction, no electron return effect, the presence of electron focusing, weaker effects on detector response and less pronounced air gap effects. In this work, the methods were developed and employed to experimentally investigate and demonstrate these properties for the first time on an inline MRI-linac system. The collected measurements were used to fine-tune and commission the radiation related aspects of the Australian MRI-linac, constituting a key step toward the application of inline MRI-linacs for patient treatments.

## Data Availability Statement

The datasets generated for this study are available on request to the corresponding author.

## Author Contributions

UJ, PK, and GL contributed to the design of this work. UJ, BD, JB, NR, and BW contributed to the acquisition of data. UJ, JB, NR, and BW contributed to the analysis and interpretation of data. UJ drafted the manuscript. All authors revised the manuscript and approved the content for publication.

### Conflict of Interest

The authors declare that the research was conducted in the absence of any commercial or financial relationships that could be construed as a potential conflict of interest.
